# Supplementary material for: Long-Term Impact of Direct-Acting Antivirals on Liver Fibrosis and Survival in HCV-Infected Liver Transplant Recipients
Source: Viruses. 2023 Aug 7;15(8):1702. doi: 10.3390/v15081702 (PMC10458217; doi:10.3390/v15081702)
Supplement: Supplementary file 1 [file viruses-15-01702-s001.zip › viruses-2485108-supplementary.pdf]

Supplementary Table S1: Adverse events occurred during the treatment period in the overall study population (n=136).

**Adverse event**	**Frequency N (%)**
Anemia	17 (13%)
Asthenia	7 (5%)
Pruritus	4 (3%)
Headache	2 (1%)
Blood pressure elevation	2 (1%)
Jaundice	1 (1%)
Dyspnea	1 (1%)
Syncope	1 (1%)
Tachycardia	1 (1%)
**Total Adverse Events**	36 (27%)
